# Need-Supportive and Need-Thwarting Teacher Behavior: Their Importance to Boys’ and Girls’ Academic Engagement and Procrastination Behavior

**DOI:** 10.3389/fpsyg.2021.628064

**Published:** 2021-03-10

**Authors:** Marie-Christine Opdenakker

**Affiliations:** Chair Group Education, University of Humanistic Studies, Utrecht, Netherlands

**Keywords:** teacher behavior, basic psychological needs, self-determination theory, academic engagement, procrastination, secondary education, differential effectiveness, gender

## Abstract

Motivation plays an important role in students’ school behavior, and research has established that students’ learning environment experiences such as teachers’ behavior toward them contribute to their motivation and behavior at school. Self-determination theory (SDT) offers an interesting frame of reference in the study of the relationship between students’ learning experiences at school and their school behavior. Considering three basic psychological needs (the need for autonomy, competence, and relatedness), the SDT points to the importance of nutriments and support in the social environment in order to allow growth in motivation, engagement, and (psychological) well-functioning. In addition, thwarting these needs is supposed to contribute to maladaptive functioning. Teachers can play an important role in the fulfillment of students’ basic psychological needs by delivering support (autonomy support, structure, and involvement); however, controlling instructional behavior, chaos in the classroom, and teacher rejection and neglect are supposed to be a treat to the fulfillment of students’ basic psychological needs. In the current innovative longitudinal study, teachers’ need-supportive behavior as well as teachers’ thwarting of these needs are considered and their relationship with students’ academic engagement (adaptive functioning) and procrastination behavior (maladaptive functioning) is studied. In addition, attention is paid to differential effects of teachers’ behavior with regard to boys and girls. Participants were 566 students belonging to 20 mathematics/English grade 1 secondary education classes in the Netherlands. Multilevel analyses revealed evidence for the importance of both teachers’ need-supportive and need-thwarting behaviors in relation to students’ academic engagement and procrastination behavior. In addition, the findings revealed that teachers’ need-supportive behavior is more important for students’ academic engagement (adaptive functioning), while teachers’ need-thwarting behavior has larger effects on students’ procrastination behavior (maladaptive functioning). Furthermore, evidence was found that boys often seemed to be more sensitive to their teachers’ behavior than girls. The findings highlight the importance of both teachers’ need-supportive and need-thwarting behaviors in daily classrooms and contribute to deepen our insight into and understanding of factors leading to adaptive and maladaptive functioning of boys and girls in relation to learning tasks at school.

## Introduction

Research has established that students’ motivation and academic engagement in school is quite important for learning, school success, and a prosperous school career (Fredricks et al., 2004; Klem and Connell, 2004; Appleton et al., 2008; Steinmayr and Spinath, 2009; Wigfield and Cambria, 2010; León et al., 2017), while procrastination behavior, which is often conceived as maladaptive behavior toward learning and school, is detrimental to academic achievements (Steel et al., 2001; van Eerde, 2003; Kim and Seo, 2015) and has negative (psychological) consequences such as experiencing guild and negative affective well-being (van Eerde, 2003). Therefore, it is important to investigate why some students are more engaged in school than others and why some students exhibit more procrastination behavior than others.

In the past, numerous studies have addressed these questions and focused, in particular with regard to procrastination behavior (see, e.g., van Eerde, 2003; Steel, 2007) but also with regard to engagement-related constructs until about a decade ago (see e.g., Guthrie and Wigfield, 2000; Blumenfeld et al., 2005), on (intra)individual student characteristics as explanations for individual differences.

However, learning environment and educational effectiveness studies as well as studies on student motivation and current motivation theories such as the self-determination theory (SDT; Ryan and Deci, 2000, 2020; Deci and Ryan, 2002) and stage–environment fit theory (Eccles and Roeser, 2009) refer to learning environment experiences (such as teacher behavior, teacher–student interaction, peer group characteristics, and interactions between peers) as important additional explanations for students’ adaptive functioning/behavior, e.g., academic engagement (Ryan and Patrick, 2001; Urdan and Schoenfelder, 2006; Roorda et al., 2011; Vansteenkiste et al., 2012; Wang and Eccles, 2012b; Stroet et al., 2013; León et al., 2017; Opdenakker, 2020) and maladaptive functioning/behavior such as procrastination, misconduct, problem, or antisocial behavior in the classroom (O’Connor et al., 2011; Vansteenkiste et al., 2012; Nordby et al., 2017; Oostdam et al., 2019; Opdenakker, 2020). Moreover, there is evidence from longitudinal studies on students’ motivation and academic engagement in school that, in general, students’ motivation and academic engagement not only decline during secondary education (Gottfried et al., 2001; Wigfield et al., 2006a,b; Skinner et al., 2008; van der Werf et al., 2008; Peetsma and van der Veen, 2011; Opdenakker et al., 2012; Wang and Eccles, 2012a) but also that students’ learning environment experiences affect (the evolution of) their motivation and academic engagement in school (Opdenakker et al., 2012; Wang and Eccles, 2012b; Stroet et al., 2015; Núñez and León, 2019). In addition, there is evidence that learning environment experiences affect the evolution of maladaptive and problem behavior as well. For example, O’Connor et al. (2011) found that the quality of teacher–student relationships was related to changes in children’s externalizing and internalizing behavior problems in school over time.

In the majority of the empirical studies on the effects of learning environment experiences on student behavior and outcomes, positive learning environment experiences (such as positive, supportive, warm teacher behavior, or teacher–student interaction) are focused on. Much less is known about the effects of negative learning environments or what is called “the dark side of teaching” (De Meyer et al., 2014, p. 541) on students’ adaptive and maladaptive behavior/functioning in relation to motivation, engagement, and procrastination since only a scarce amount of empirical research addresses the effects of “the dark side of teaching.” There are some indications in the literature on the quality of teacher–student relations in kindergarten and elementary school (see, e.g., some contributions in Wubbels et al., 2012) that low-quality and conflicting relationships and low responsiveness of teachers toward children are associated with students’ problem behavior. In addition, Roorda et al. (2011), reviewing the influence of affective teacher–student relationships on students’ academic engagement (from preschool to high school) using a meta-analytic approach, found evidence for medium to large associations between negative (and positive) relationships and (academic) engagement. Furthermore, in a recent study of Vandenkerckhove et al. (2019) in which the relation between weekly need-based experiences (based on experiences with teacher, school, and peers) and weekly academic (mal)adjustment was investigated, positive relations were found between weekly variations in need satisfaction and weekly variations in positive affect, engagement, and autonomous motivation and between variations in need frustration and variations in negative affect, disaffection, and controlled motivation.

Research on the relation between the extent to which teachers meet the basic psychological needs of their students and maladaptive student behavior or maladaptive functioning at school (or disengagement or disaffect) is even more scarce and has been studied by only a handful of researchers (e.g., Ryan and Patrick, 2001; Skinner et al., 2008; Vansteenkiste et al., 2012; Gunnell et al., 2013; Koerhuis and Oostdam, 2014; Jang et al., 2016; Oostdam et al., 2019; Vandenkerckhove et al., 2019; Nouwen and Clycq, 2020).

It is also striking that there is relatively little research that pays attention to differential effects of teacher behavior in relation to student gender on student adaptive (e.g., academic engagement) and maladaptive behavior (e.g., problem behavior, procrastination, etc.), although gender differences with regard to adaptive and maladaptive behavior are studied and recognized. One seems to assume that what is good for boys is also good for girls. On of the exceptions is the study of Koerhuis and Oostdam (2014), in which evidence for differential effects of basic needs satisfaction by the teacher on boys’ and girls’ problem behavior was found. In the limited number of studies focusing on gender differences related to the effects of teacher behavior, coaching, and learning environment characteristics (e.g., Van de Gaer et al., 2006; Lietaert et al., 2015; Hughes and Coplan, 2018), there is some evidence that gender has a moderating effect, indicating a greater sensitivity of boys.

In the present study, we will address the mentioned gaps while adopting the comprehensive theoretical framework of the SDT (Ryan and Deci, 2000, 2020; Deci and Ryan, 2002), and in particular the central mini-theory basic psychological need theory (BPNT; Vansteenkiste et al., 2020), to study the association between need-supportive and need-thwarting teacher behavior and students’ academic engagement and procrastination behavior. As such, we embed academic engagement and procrastination behavior within a (broader) motivational framework. This gives the opportunity to theorize both concepts in relation to underlying psychological processes and ongoing interactions with the learning and social context (interaction with teachers and their behavior). Support for this approach can be found, in any case, in relation to academic engagement, in the works of Skinner and Belmont (1993), Skinner et al. (2008), Wang and Fredricks (2014), and Reeve and colleagues (see, e.g., in Jang et al., 2016) and is in agreement with the self-system model of motivational development (Connell and Wellborn, 1991), a model grounded in the SDT, and with the idea of the dual-process model with a SDT framework as described by Jang et al. (2016).

In addition, arguments for this approach are that academic engagement and procrastination behavior can change *via* cyclic interactions with contextual factors (such as learning environment characteristics, and teacher behavior) and influence later academic, behavioral, and social student outcomes, which are the products of these context-influenced changes in engagement and procrastination behavior. Evidence for this can be found in work of Jang et al. (2016).

Up till now, the SDT framework (or the related self-system model of motivational development) has been adopted by several scholars in engagement research, but not yet in procrastination research. However, the importance of a motivational perspective is already recognized by some procrastination researchers (see, e.g., Rozental and Carlbring, 2014; Steel et al., 2018) and also in research on problem behavior in general. Recently, a few scholars investigating student problem behavior adopted the SDT framework (e.g., Oostdam et al., 2019).

In the next section, the SDT framework will be introduced as the theoretical framework of this study, followed by an introduction into the conceptualization of academic engagement and procrastination and a discussion of research findings concerning associations with learning environment characteristics and, in particular, teacher behavior. Because research on teacher behavior–procrastination associations is very scarce, the perspective will be somewhat broadened to associations between teacher behavior and maladaptive student behavior with regard to learning and/or in school.

## Theoretical Background

### Self-Determination Theory and the Importance of Satisfying the Needs of Autonomy, Competence, and Relatedness

Self-determination theory (Ryan and Deci, 2000, 2020; Deci and Ryan, 2002), and in particular the BPNT (Vansteenkiste et al., 2020), offers an interesting frame of reference in the study of the relationship between students’ learning experiences at school and their school behavior. SDT asserts that every person, irrespective of his culture, requires the fulfillment of three fundamental innate psychological needs in order to function well, to flourish, and to experience psychological growth and well-being (Ryan and Deci, 2000). These needs are the need to feel autonomous, the need to feel competent, and the need to feel related. BPNT considers both the satisfaction and frustration of these needs, “with frustration representing a stronger and more threatening experience than the mere absence of its fulfillment” (Vansteenkiste et al., 2020, p. 3).

According to SDT–BPNT (Ryan and Deci, 2000, 2020; Deci and Ryan, 2002; Vansteenkiste et al., 2020), the need to feel autonomous means that individuals are born with the need to act in congruence with their true selves and express their genuine preferences in order to experience a general sense of choice, volition, willingness, and ownership. When this need is satisfied, a sense of integrity is experienced “as when one’s actions, thoughts, and feelings are self-endorsed and authentic” (Vansteenkiste et al., 2020, p. 3). When this need is frustrated, one experiences pressure, external control, conflict, or feeling pushed in a direction that one does not want (Ryan and Deci, 2020; Vansteenkiste et al., 2020). The need to feel competent indicates that individuals are born with the need to experience themselves as effective in their interactions with the (social) environment, to feel a sense of mastery and have opportunities to express and extend their abilities (Deci and Ryan, 2002). When this need is frustrated, feelings of personal ineffectiveness, failure, or helplessness are experienced (Vansteenkiste and Ryan, 2013; Vansteenkiste et al., 2020). The need to feel related indicates that individuals are born with an innate desire to emotionally connect to others (Skinner and Pitzer, 2012), to feel loved and cared for, and to feel a sense of belonging in a particular enterprise (Baumeister and Leary, 1995). Relatedness refers to experiencing warmth, care, and bond and, when frustrated, having “a sense of social alienation, loneliness, and exclusion” (Vansteenkiste et al., 2020, p. 3). It is important to realize that within SDT-BPNT, need frustration is considered to be distinct from the absence of need satisfaction (Vansteenkiste et al., 2020).

Considering the three basic psychological needs, SDT-BPNT points to the importance of nutriments and support in the social environment in order to allow growth in motivation, engagement and (psychological) well-functioning (Deci and Ryan, 2002), and flourishing (Vansteenkiste et al., 2020). In addition, thwarting these needs, which leads to need frustration (Vansteenkiste and Ryan, 2013; Vansteenkiste et al., 2020), is supposed to contribute to maladaptive functioning (Ryan and Deci, 2000), such as disengagement, disaffection, and exhibiting problem behavior. In the past, need thwarting was originally conceived as the opposite pole of need-support, but recently, SDT researchers have begun to see/study need-supportive and need-thwarting “opposites” as separate dimensions (Reeve et al., 2014), and it is recognized that little support for the needs leads to experiences of low (i.e., deprived) need satisfaction, while a more direct thwarting of individuals’ needs will lead to experiences of need frustration (Ryan and Deci, 2017). In addition, it is hypothesized that psychological need frustration will, compared to need deprivation, more strongly lead to maladjustment because it represents a more direct and stronger thread to individuals’ need-based functioning (Vansteenkiste and Ryan, 2013).

In schools, the major function of students’ basic psychological needs is, according to SDT, to vitalize their inner motivation, which in turn facilitates their engagement in learning and classroom activities (Niemiec and Ryan, 2009; Molinari and Mameli, 2018). Teachers can play an important role in the fulfillment of students’ basic psychological needs by creating a supportive learning environment and delivering support (autonomy support, structure, and involvement). For example, teachers can give students meaningful choices and tasks, attempt to understand, acknowledge, and respect students’ perspective, and give them a voice and a meaningful rationale as things have to be done (autonomy support). In addition, teachers can create a supportive well-structured class environment in which there are consistent guidelines and rules and clear expectations and goals so that students know what it takes to do well in class, in which students receive help and informational and instructional support when they need it, students can achieve success and feel competent, and where optimal challenges, positive and efficacy supportive feedback, and opportunities for growth are afforded (structure). Lastly, it is important that teachers create a caring, respectful, and supporting environment that meets students’ need for relatedness (teacher involvement) (Ryan and Deci, 2020). However, controlling instructional behavior, chaos in the classroom, uncertainty, inconsistent teacher behavior, and teacher rejection and neglect are supposed to be a treat to the fulfillment of students’ basic psychological needs and can be seen as thwarting students’ basic psychological needs.

Next to this, SDT also provides an integrated conceptualization for the internalization of external demands (Appleton et al., 2008), which is quite important in the context of learning at school. SDT acknowledges that the catalyst for behavior in many situations is external to oneself (Ryan and Deci, 2000) and specifies qualitative differences in the level of self-determination associated with extrinsic motivation (Ryan and Deci, 2020). SDT argues “that need supports enhance intrinsic motivation and internalization, resulting in higher achievement, whereas, paradoxically, attempting to control achievement outcomes directly through extrinsic rewards, sanctions, and evaluations generally backfires, leading to lower-quality motivation and performance” (Ryan and Deci, 2020, pp. 1–2). In addition, SDT predicts that greater internalization (and competence) is facilitated by high levels of both autonomy support and provision of structure (Jang et al., 2010; Ryan and Deci, 2020). However, to fully understand the variability in the process of internalization, SDT also includes the need for relatedness (Vansteenkiste et al., 2020) and posits the relevance of supporting (or thwarting) this need. In the internalization process, values and regulations are “taken in” and non-intrinsically motivated behaviors can become self-determined; it concerns, for example, in a school context, the internalization of the regulation of positive school-related behaviors (Ryan and Deci, 2000). So, ideally, the social context needs to satisfy all three basic psychological needs to foster the process of internalization (Milyasvkaya et al., 2014) and internalization is hampered if one of the needs is frustrated. This means that experiencing a strong bond with the teacher (and feeling effective carrying out a non-interesting learning task) may provide a good starting point to begin the internalization, but the fulfillment of autonomy is additionally needed for full internalization. In other words, the internalization process will be only partial when the need for autonomy (p. 5) remains unfulfilled.

There is considerable evidence for the importance and relevance of SDT in a variety of domains, including education, linking the effects of social contexts (autonomy support, structure, and involvement) to basic needs satisfaction and a variety of student/individual outcomes (for reviews, see, e.g., Ryan and Deci, 2000, 2020; Deci and Ryan, 2002; Vansteenkiste et al., 2020). However, psychological need thwarting, which can be considered as a feeling that arises in response to an individual’s perception that his/her psychological needs are actively undermined by others (Bartholomew et al., 2011), is an understudied component of SDT (Costa et al., 2015). From the few studies that addressed this topic, evidence is found for the relevance of need thwarting in relation to maladaptive functioning (see, e.g., Bartholomew et al., 2018; Vandenkerckhove et al., 2019) or as a mediator between context characteristics and maladaptive functioning (Hein et al., 2015; Bashir et al., 2019). In these studies, evidence was found for the negative effects of need frustration or controlling behavior in the social context.

### Academic Engagement and Associations With Teacher Behavior

In general, academic engagement (sometimes also named school, classroom, or student engagement) reflects, among other things, a student’s active involvement in a task or activity related to school (Reeve et al., 2004). Some researchers refer to “energy in action” (Ainley, 2012; Skinner and Pitzer, 2012). Marks (2000, pp. 154–155) defines engagement as “a psychological process, specifically, the attention, interest, investment, and effort students expend in the work of learning.” In the literature on engagement, considerable differences exist with regard to the conceptualization of this construct as well as with regard to its operationalization and measurement (Fredricks et al., 2004; Appleton et al., 2008). However, one constant across the myriad conceptualizations of engagement is that it is, nowadays, by most researchers seen as a multidimensional or multifaced construct including two to four components, sometimes also labeled as “dimensions,” “subtypes,” or “facets” (Appleton et al., 2008; Voelkl, 2012; Wang and Fredricks, 2014). Most definitions contain behavioral components, and many also include emotional/affective/psychological components. Academic or cognitive components are sometimes included as well, and recently, some scholars (e.g., Reeve and Tseng, 2011; Reeve, 2012, 2013; Molinari and Mameli, 2018) make a plea to also include an agency component. Examples of the behavioral components are participation in school-related activities, involvement in learning and academic tasks, and positive conduct. Emotional/affective/psychological components refer to interest, positive attitude toward learning, and values related to positive or negative interactions with teachers, classmates, academics, and school (including, identification, belonging, etc.). According to Fredricks et al. (2004), this component is “presumed to create ties to an institution and influence willingness to do the work” (p. 60). Cognitive components refer to using processes and strategies to elaborate contents that need to be learned; it includes learning goals, self-regulation, and (psychological) investment in learning. Academic components refer to time on task, homework completed, etc. Agentic engagement, initially proposed by Reeve and Tseng (2011) and investigated by, e.g., Reeve (2013), is described by Reeve (2012, p. 161) as “the process in which students proactively try to create, enhance, and personalize the conditions and circumstances under which they learn.” So, it emphasizes students’ active role and transformative contribution to their learning conditions and circumstances, which is in contrast to the other components of engagement referring to the reactions of students toward school activities and tasks as a whole.

In a lot of research on academic engagement, engagement is defined based on the tripartite conceptualizations of Fredricks et al. (2004) or on the motivational conceptualization with two dimensions of engagement of Skinner et al. (2008). The conceptualization of Fredricks et al. (2004), which was based on a review of the engagement literature, approaches academic (school) engagement as a “meta construct” distinguishing between behavioral engagement (referring to participation and involvement in activities), emotional engagement (referring to encompassing both positive and negative reactions to teachers, classmates, academics, and school influencing willingness to work), and cognitive engagement (referring to the investment in learning and willingness to put in effort necessary to comprehend complex ideas and master difficult skills). In the definition of Skinner et al. (2008), engagement is motivationally conceptualized and refers to “students’ active participation in academic activities in the classroom” (Skinner et al., 2008, p. 766). Skinner and colleagues distinguish between a behavioral and an emotional dimension. The behavioral dimension of engagement includes “students’ effort, attention, and persistence during the initiation and execution of learning activities” (Skinner et al., 2008, p. 766), while the emotional dimension “focuses on states that are germane to students’ emotional involvement during learning activities such as enthusiasm, interest, and enjoyment” (Skinner et al., 2008, p. 766). There is some evidence that the dimensions of engagement are moderately correlated. For example, Blumenfeld et al. (2005) found correlations between 0.52 and 0.60 for behavioral, cognitive, and emotional engagement.

In general, despite the different conceptualizations, there is a growing interest in students’ engagement from a scientific and practical point of view (Molinari and Mameli, 2018). One of the main reasons is that, nowadays, engagement is presumed to be malleable (Fredricks et al., 2004), being the resultant of an interaction between the individual and the context, and therefore, it is responsive to variations in environments (Blumenfeld et al., 2005). Engagement is considered to operate as a protective factor for motivational problems, problem behavior, and alienation from school (Li and Lerner, 2011; Wang and Fredricks, 2014) and, therefore, crucial to the amelioration of students’ educational paths (Appleton et al., 2008).

While current research on students’ engagement for school often has the student in context as the primary focus of study, earlier work focused almost entirely on individual differences (Blumenfeld et al., 2005). One of findings often found in these studies (and still in some more recent studies) was the difference in engagement between boys and girls, indicating a higher (behavioral and emotional) engagement of girls (Marks, 2000; Fredrickx et al., 2005; Wang et al., 2011).

Skinner et al. (2008) explored students’ behavioral and emotional engagement and found that behavioral engagement, which is often seen as “the most prototypical of engagement” (Skinner et al., 2008, p. 778), has the lowest cross-time stability and the fastest drop across the school year of the two. Based on their research findings, they concluded that behavioral engagement seemed to be “a good summary indicator, diagnostic of the state of the entire motivational system” (Skinner et al., 2008, p. 778). However, they also mentioned that emotional engagement, which is a bit more stable than behavioral engagement, “seems to be a sensitive barometer of the whole motivational system” and “the active ingredient in sustaining motivation.” In addition, they found that emotional and behavioral engagement was shaped over time by the fulfillment of basic psychological needs (especially strong contributions from feeling autonomous) and also by students’ perceptions of their teachers’ support (involvement, structure, and autonomy support). In addition, Vandenkerckhove et al. (2019), exploring the associations between weekly variations in need satisfaction and motivational outcomes (engagement, autonomous motivation, and positive affect), found evidence for positive relations. Also, the research of Zimmer-Gembeck et al. (2006) found evidence for the positive effects of learning environments in which the principles of basic need satisfaction are met (relationships with teachers and peers) on academic engagement.

Numerous studies found evidence for the importance of teacher support (often conceptualized as a mixture of academic and interpersonal support) to students’ (academic) engagement (Connell and Wellborn, 1991; Skinner and Belmont, 1993; Marks, 2000; Fredricks et al., 2004; Klem and Connell, 2004; Blumenfeld et al., 2005; Eccles and Roeser, 2011; Wang and Eccles, 2012b; Stroet et al., 2013; Wentzel, 2016; León et al., 2017; Opdenakker, 2020), and various studies have shown that teachers’ ability to meet students’ basic psychological needs correlates with students’ engagement (Taylor et al., 2010). Ryan and Patrick (2001) operationalized teacher support as the promotion of good teacher–student and peer relationships and found that students’ perception of teacher support was related to positive changes in engagement, while students’ perception of the teacher as promoting performance goals (competition and comparison) was related to negative changes in engagement. Teachers who are caring, trusting, and respectful toward students provide them the socioemotional support that they need to approach, engage in, and persist with academic learning tasks (Wentzel and Wigfield, 2007; Hattie, 2009; Wigfield et al., 2015). Further evidence for the importance of good/positive teacher–student relationships, teacher involvement, or the degree to which the teacher is able to meet students’ need for relatedness can be found in the studies of, for example, Connell and Wellborn (1991), Rosenfeld et al. (2000), Tucker et al. (2002), Furrer and Skinner (2003), Brewster and Bowen (2004), Daly et al. (2009), Murray (2009), Wang and Eccles (2012b), and Nouwen and Clycq (2020), and in the review study of Osterman (2000).

In addition, studies have shown that the relation between the quality of teacher–student relationships (or teacher involvement toward students) and engagement is reciprocal over time (Skinner and Belmont, 1993; Ladd et al., 1999; Skinner and Pitzer, 2012), indicating that high-quality teacher–student relationships have a positive effect on students’ basic need satisfaction and engagement, which in turn elicits further teacher support (Skinner and Pitzer, 2012). Unsupportive and conflictual teacher–student interactions and relationships have the opposite effect and lead to even further withdrawal of teacher support.

Evidence for the importance of structure and well-managed classrooms for students’ academic engagement is also found (Connell and Wellborn, 1991; Skinner and Belmont, 1993; Tucker et al., 2002; Nie and Lau, 2009; Pianta et al., 2012) and, in line with this, for satisfying the need to feel competent. For example, Molinari and Mameli (2018) found a positive association with behavioral, cognitive, and emotional engagement. For several components of structure such as clarity and guidance, positive associations are found as well. For an overview, see Stroet et al. (2013).

Various studies, e.g., the work of Reeve et al. (2004), also showed that autonomy support predicts engagement (and that teachers can be successfully trained to deliver autonomy support) (Reeve et al., 2004). However, Skinner and Belmont (1993) failed to document a link between perceived autonomy support and engagement, while the study of Skinner et al. (2008) revealed that students’ self-perceptions of especially autonomy (in addition to competence and relatedness) contributed to changes in behavioral engagement. In other studies, evidence was also found for a positive association between autonomy support and engagement (Tucker et al., 2002; Shih, 2008). Assor et al. (2002) found that fostering relevance (a component of autonomy support) and showing disrespect (a component of autonomy thwarting) were uniquely respectively positively and negatively associated with students’ engagement. In the studies of Jang et al. (2010, 2016), a positive relation between observed/perceived autonomy support and engagement was found, and the study of Núñez and León (2019) showed that perceived autonomy support was a significant predictor of the need for autonomy, which, in turn, predicted changes in (four types of) students’ engagement.

Several studies paid attention to the unique contribution of each of the teacher support dimensions, and although most of the time strong relations were found with (one or more dimensions of) engagement, evidence concerning the unique contributions of the teacher support dimensions is mixed. For example, Skinner and Belmont (1993) found that only students’ perceptions of structure had unique effects on behavioral engagement, while students’ perceptions had only unique effects on emotional engagement, and Murray did not find evidence for unique effects. However, Furrer and Skinner (2003) found that relatedness (referring to social partners, including teachers) mattered beyond perceived control. Jang et al. (2010) investigated the effect of (observed) autonomy support and structure and found evidence that both are relevant. Nie and Lau (2009) found evidence for unique effects of structure and affect (an aspect of involvement). Tucker et al. (2002) and Skinner et al. (2008) found evidence for unique contributions of all three support dimensions. In addition, tests of process models in the study of Skinner et al. (2008) revealed that the effects of teacher behavior were mediated by children’s self-perceptions of need satisfaction.

Only a few studies paid attention to possible differential effects of the mentioned dimensions of teacher support. One example is the study of Furrer and Skinner (2003). Their study indicated that, although girls experienced, on average, higher levels of relatedness, the effect of perceived relatedness (especially with teachers) on engagement was more salient for boys. However, Skinner et al. (2008) did not find such differential effects of teacher support.

### Academic Procrastination, Maladaptive Behavior, and Associations With Social Context/Teacher Factors

Academic procrastination can be defined as postponing, delaying, or putting off a task or a decision related to learning or school despite its given priority. In the literature on procrastination, a variety of definitions can be found, but all conceptualizations recognize the existence of postponing, delaying, or putting off a task or a decision (Steel, 2007). However, in order to classify that behavior as procrastination, the task or decision should also be irrationally and needlessly delayed despite its given priority (Silver and Sabini, 1981). In line with this, Steel (2007) defines procrastination as a form of self-regulatory failure, where one “voluntarily delay[s] an intended course of action despite expecting to be worse off for the delay” (Steel, 2007, p. 66), indicating that the core characteristic of procrastination is the intention–action gap. This means that the problem is not a lack of good intentions, but that, too often, intentions are not implemented as planned (Dewitte and Lens, 2000). van Eerde (2000) refers to procrastination as “the avoidance of the implementation of an intention” (p. 374). Others, e.g., Schraw et al. (2007), define academic procrastination as a purposive delay of academic tasks that must be completed. Reviewing the literature on procrastination of the last two decades, it becomes clear that procrastination is more and more seen as a self-regulation failure (Pychyl and Flett, 2012), and a growing body of evidence for this view is found (e.g., in the longitudinal study of Ziegler and Opdenakker, 2018).

There is some evidence from meta-analyses and epidemiological studies that procrastination is (weak) negatively associated with age (indicating somewhat more procrastination at younger ages) and with gender (indicating that boys tend to procrastinate somewhat more than do girls) (van Eerde, 2003; Steel and Ferrari, 2013). No evidence is found for an association with intellectual ability (van Eerde, 2003; Rozental and Carlbring, 2014), but moderate associations are found in meta-analyses/reviews with intrapersonal factors such as personality (e.g., consciousness) (van Eerde, 2003; Steel, 2007), impulsiveness (Steel, 2007), self-image (van Eerde, 2003), self-efficacy (Steel, 2007), motives, e.g., perfectionism (van Eerde, 2003), achievement motivation (Steel, 2007), affect (e.g., state anxiety) (van Eerde, 2003), and other psychological variables such as task avoidance (Ferrari et al., 1995) and task aversiveness (Steel, 2007). Recent studies on academic procrastination found evidence for self-esteem (Chen et al., 2016), self-efficacy (Wolters, 2003; Klassen et al., 2008; Corkin et al., 2011), self-control (Gustavson et al., 2014; Rozental and Carlbring, 2014), achievement motivation (Saddler and Buley, 1999), and time perspective (Chen and Chang, 2016; Chen and Kruger, 2017), and in a recent longitudinal study on academic procrastination in secondary education (Ziegler and Opdenakker, 2018), a clear (and stable) negative association was detected with effort regulation, as well as somewhat weaker negative associations with metacognitive self-regulation and self-efficacy, which became weaker over time as the school year progressed.

Within traditional procrastination research, procrastination is often regarded as a character trait or behavioral disposition that remains stable across time and contexts (e.g., Schouwenburg and Lay, 1995; Lay, 1997; van Eerde, 2000). However, a growing body of literature is coming to the front that points to the dynamic nature of procrastination, suggesting that changes in procrastination behavior over time may occur due to contextual and task-related factors (Senécal et al., 1997; Blunt and Pychyl, 2000; Wolters, 2003; Moon and Illingworth, 2005; Steel, 2007; Wäschle et al., 2014; Chen and Han, 2017; Nordby et al., 2017; Steel et al., 2018). The already mentioned study of Ziegler and Opdenakker (2018) is in line with this view. In this study, evidence was found for the dynamic nature of academic procrastination of grade 7 students indicating a linear increase of procrastination behavior during their first year in secondary education.

Studies on the effects of contextual and social environment factors in relation to procrastination behavior are, surprisingly, scarce (Corkin et al., 2014; Chen and Han, 2017; Nordby et al., 2017), but highly needed, as van Eerde (2003) already mentioned in her review study. She found, for the majority of categories distinguished in her meta-analytic study, heterogeneity of the effect sizes, indicating room for moderator effects (of such factors). It is quite striking that contextual and social environment factors have received considerably less attention than individual factors given the fact that being a student is an inherently social endeavor, as noted by Nordby et al. (2017). Of the small amount of studies investigating the associations between contextual/social factors and academic procrastination, two addressed peer influences (Chen et al., 2016; Nordby et al., 2017), one social support networks of friends and family (Ferrari et al., 1998), one ecological assets (referring to support from family, empowerment, boundaries and expectations, and constructive use of time, e.g., by being involved in a religious group/church) (Chen and Han, 2017), and a few addressed teacher/instructor and class climate effects on students’ academic procrastination. Related to these studies, evidence was found for negative associations between students’ procrastination and instructor support and organization (Corkin et al., 2014), of which the first (i.e., support) referred to expressing warmth and a personal interest in their students and providing academic assistance and the second (i.e., organization) referred to whether the course content, instructor expectations, deadlines, and evaluation criteria were perceived by the students as clear, which is related to structure support in a SDT perspective. These instructor factors were thought to make it easier for students to “organize, structure, and plan their own work” (Corkin et al., 2014, p. 299). This line of reasoning is supported by the mediation of this instructor effect by self-efficacy, which is in line with the temporal motivation theory of Steel (2007). According to this theory, motivation diminishes and procrastination becomes more likely when students do not expect to meet the competence standards of a given task, i.e., when they do not feel very self-efficacious. Similar results were reported in a qualitative study indicating that unorganized and lax teachers were a reason to procrastinate (Grunschel et al., 2013). Another teacher factor that has been studied is related to the expectations teachers hold for their students. There is some evidence indicating that holding low expectations for students promotes procrastination (Schraw et al., 2007).

Up till now, to my knowledge, no studies exist relating academic procrastination to the dimensions of basic psychological needs or to the degree to which teachers create environments that satisfy these needs. However, a handful of studies exist relating maladaptive behavior/functioning at school to these dimensions. Examples are the study of Ryan and Patrick (2001), Vansteenkiste et al. (2012), Koerhuis and Oostdam (2014), Hein et al. (2015), and Oostdam et al. (2019). In all these studies, evidence is found for the importance of teachers in relation to students’ maladaptive behavior/functioning at school. The extent to which teachers meet the satisfaction of the basic psychological needs of students is negatively related to the maladaptive behavior/functioning of students. For example, the study of Vansteenkiste et al. (2012), investigating the effects of teaching configurations with differences in the amount of autonomy support and structure, revealed that students who perceived their learning environment as offering low autonomy support and less structure showed, on average, more problem behavior in comparison to students who perceived their learning environment as highly autonomy supportive (irrespective of the amount of structure in it). This indicates that, in particular, the creation of an autonomy-supportive learning environment seems to be relevant with regard to reducing problem behavior. Hein et al. (2015) found that components of controlling teacher behavior (negative conditional regard and intimidation) had a significant indirect effect on students’ bullying behavior and feelings of anger through perceived psychological need thwarting. Nouwen and Clycq (2020) found evidence for the importance of teacher involvement having direct and indirect effects (*via* the satisfaction of the need to feel related, autonomous, and competent) on school misconduct (non-compliance to school regulations). The study of Oostdam et al. (2019) addressed the relation between the extent to which teachers met the three basic psychological needs of their students and the maladaptive behavior of their students. They found a significant negative relation indicating that the more the teacher met the needs of their students, the less problem behavior (e.g., unfriendly behavior) was present. In addition, their research revealed that the extent to which the teacher met their students’ needs became increasingly important over time. Koerhuis and Oostdam (2014) discovered differential effects of teacher support indicating that the problem behavior of boys was in particular associated with the degree to which their teachers satisfied their need to feel autonomous, while for girls, the need to feel related mattered most.

In addition to these studies on maladaptive behavior, several studies address disengagement or disaffection as a form of maladaptive functioning. Examples are the studies of Skinner et al. (2008), Jang et al. (2016), and Vandenkerckhove et al. (2019). Disaffection refers to “the occurrence of behaviors and emotions that reflect maladaptive motivational states.” It has a behavioral component, “including passivity and withdrawal from participation in learning activities,” and an emotional component, “including boredom, anxiety, and frustration in the classroom” (Skinner et al., 2008, p. 767). Skinner et al. (2008) found evidence that teacher support (based on the three dimensions of SDT) and students’ self-perceptions (especially autonomy) contributed to changes in behavioral disaffection. For emotional disaffection, however, autonomy was the only significant predictor of the declines. Despite robust concurrent correlations, neither relatedness nor perceived control was a significant predictor of decreases in emotional disaffection. Furthermore, student reports of teacher support predicted declines in behavioral and emotional disaffection over time. Vandenkerckhove et al. (2019) discovered that weekly variations in need frustration related positively to weekly variations in disaffection. Jang et al. (2016) adopted a dual-process model with a SDT framework and studied the effects of teachers’ autonomy support and control, need satisfaction, and frustration on (dis)engagement, defining disengagement as a multidimensional construct entailing behavioral, emotional, cognitive, and agentic components. They found—with regard to disengagement—that students’ perceived teacher control predicted longitudinal changes in need frustration which predicted changes in disengagement, indicating that a trajectory of rising disengagement emerged out of the darker side processes of perceived teacher control and need frustration. In addition, they discovered that high levels of disengagement seemed to lead to a trajectory of rising control and falling autonomy support according to student perceptions. Frenzel et al. (2007) discovered relationships between perceived teacher behavior (instructional support and being disrespectful) and students’ emotional experiences related to disaffection (boredom and anxiety).

## Aim of the Present Study

The present study investigates whether need-supportive and need-thwarting teacher behaviors (as perceived by students) are related to students’ academic engagement and procrastination behavior, which can be respectively conceived as a form of adaptive and maladaptive student behavior. In addition, the possibility of differential effects of need-supportive and need-thwarting teacher behaviors in relation to student gender was explored. By paying attention to need-supportive as well as need-thwarting behavior within the same study, exploring differential effects of these kinds of teacher behavior in relation to student gender and relating these kinds of teacher behavior to forms of adaptive as well as maladaptive student behavior, while adopting a longitudinal approach, the study extends the existing research.

Based on the SDT, and in particular the basic psychological needs theory (and in agreement with the self-system model of motivational development), it is expected that the different dimensions of need-supportive and need-thwarting teacher behaviors are associated with students’ academic engagement and procrastination behavior. In line with the (small amount of) literature on differential effects of learning environment characteristics in relation to student gender, it is expected that, if differential effects will be found, they will indicate a higher sensitivity of boys toward the behavior of their teachers.

## Materials and Methods

### Participants

Participants were 566 students (45% girls, 55% boys) belonging to 20 mathematics/English grade 7 secondary education classes of three public schools in the Netherlands. Classes were obtained with stratified sampling. The three schools they belonged to were located in a provincial city area in the northern part of the Netherlands. The schools were representative of typical public schools for middle socioeconomic status. The class sizes ranged from 21 to 31 students. Half of the classes were math classes. Math and English classes were chosen because these are important and diverse subjects in grade 7 and because it was expected that choosing these classes would result in heterogeneous teacher behavior. For both subjects, classes of all school tracks of the regular Dutch education system were represented, including the so-called transition classes (that combined several track levels in one class, 40%) as well as single-track classes (prevocational, general, and pre-university). The mean age of the students was 12.19 years (SD = 0.55), and less than 1% of the students were nonnative Dutch.

### Procedure

Several paper-and-pencil questionnaires were used to tap students’ perceptions of teachers’ need support and need thwarting during the first months of the school year and their (subject-related) engagement and procrastination behavior (at the start of the school year and after about 2 months).

Upon receiving the school authority’s permission—which was based on voluntary participation in the research—and written informed consent from the students’ math/English teachers and their parents/representatives, the questionnaires were distributed during class time. The students completed the questionnaires after having the purpose of the research explained to them. They were assured of their confidentiality and anonymity, and in order to assure this, the administration of the questionnaires at the different time points was carried out by research assistants.

### Measures

#### Need-Supportive and Need-Thwarting Teacher Behavior

Students’ perceptions of teacher support and teacher thwarting were measured by means of the Questionnaire on Teacher Support and Thwart. This questionnaire is based on the Teacher as Social Context (TASC; Belmont et al., 1992) and contains 51 items. The items relate to teacher support (autonomy, structure, and teacher involvement), omission of support, supposed opposites like controlling instructional behavior, chaos, uncertainty, and inconsistency in the classroom, and teacher neglect/rejection, resulting in six scales. Examples of the items are: “My teacher gives me a lot of choices about how I do my schoolwork,” “This teacher tries to control everything I do,” “This teacher shows how to solve problems for myself,” “My teacher keeps changing how he/she acts towards me,” “This teacher really cares about me,” and “My teacher doesn’t seem to enjoy having me in his/her class.” For convenience, we will refer to the dimensions/scales as autonomy support *versus* teacher thwart—control, structure versus teacher thwart—chaos/inconsistency, and teacher involvement *versus* teacher thwart—neglect/rejection. The number of items of the six individual teacher behavior scales ranges from 5 to 12, and the psychometric properties of the individual scales are sufficient to good (Cronbach’s α values vary between 0.61 and 0.82). Items were presented on a five-point Likert scale ranging from 1 = “strongly disagree” to 5 = “strongly agree.”

#### Academic Engagement

Students’ self-reported academic engagement was measured by a frequently used scale in Dutch scientific research based on the work of Roede (1989) and refers to engaged behavior and emotion. The content of the scale is in line with the concept of (behavioral) engagement of Skinner et al. (2008, 2009), which Skinner et al. (2008) describe as “a good summary indicator, diagnostic of the state of the entire motivational system” (p. 778). The scale consists of three (start) or five items (end). Examples of items are: “For this subject I do my best during the lessons” and “For this subject I enjoy working for school.” The reliability of the scale scores (Cronbach’s α) ranges from 0.65/0.63 (math/English start) to 0.86/0.83 (math/English end). Items were presented on a five-point Likert scale ranging from 1 = “strongly disagree” to 5 = “strongly agree.”

#### Procrastination Behavior

Students’ self-reported procrastination was based on the procrastination scale of Depreeuw and Lens (1998) and refers to postponing planned activities and frequent failure at doing what ought to be done to reach goals in relation to schoolwork. An example item is “I start studying later than I had intended to.” The scale consists of five items and the reliability of the scale scores (Cronbach’s α) ranges from 0.65/0.77 (math/English start) to 0.81/0.83 (math/English end). Items were presented on a five-point Likert scale ranging from 1 = “strongly disagree” to 5 = “strongly agree.”

### Method of Analysis

Multilevel analyses (MLwiN; Rasbash et al., 2012) were performed to study the effects of the need-supportive and need-thwarting dimensions of teacher behavior on the differences (and changes) in student engagement and procrastination behavior. In the multilevel models, two levels were distinguished, namely, the class level (classes) and the student level (students within classes). In addition, a control for prior academic engagement/procrastination behavior (measured at the start of the school year) was carried out. Furthermore, models with and without student gender and student gender–teacher behavior dimension interactions were inspected. Models including teacher behavior, student gender, and the interaction between student gender and teacher behavior inform about the evidence for differential effects of teacher behavior in relation to student gender or, otherwise stated, inform about the differential sensitiveness of boys/girls in relation to teacher behavior. In addition, hierarchical models with and without a combination of need-supportive and need-thwarting teacher behaviors were inspected as well in order to explore evidence for unique and joint effects of these teacher behavior dimensions. Finally, models with student gender and the interaction between student gender and teacher behavior included were analyzed twice: firstly, with student gender coded as 0 = boy and 1 = girl and, secondly, with 0 = girl and 1 = boy in order to get the whole picture with regard to the differential sensitiveness of boys and girls in relation to teachers’ need-supportive and need-thwarting behaviors and especially as an optimal estimation of the effects of teachers’ behaviors for both boys and girls. The results in the tables are, in accordance with usual practice, presented with significance levels referring to two-sided testing. However, based on the literature/theoretical framework (and expectations derived from it), one-sided testing is allowed with regard to the effects of teaching behavior and the interaction between student gender and teaching behavior.

## Results

### Descriptive Statistics

The descriptive statistics of all variables (means and standard deviations) are provided in Table 1.

**TABLE 1:** Means (*M*) and standard deviations (SD) for teacher support and teacher thwart dimensions, student academic engagement, and student procrastination behavior.

	*M*	SD
**Support and thwart dimensions**		
Autonomy support	3.20	0.70
Teacher thwart—control	2.04	0.62
Structure	3.48	0.67
Teacher thwart—chaos/inconsistency	1.95	0.58
Teacher involvement	3.02	0.64
Teacher thwart—neglect/rejection	1.70	0.66
**Student outcomes**		
Academic engagement (start)	3.87	0.80
Academic engagement (end)	3.68	0.85
Procrastination (start)	2.09	0.83
Procrastination (end)	2.22	0.88

As can be seen in the table, students in grade 7 math/English classes perceive, on average, more need-supportive than need-thwarting teacher behavior, and of the need-thwarting behaviors, they experience most controlling teacher behavior and chaos/inconsistency. With regard to need-supportive teaching behavior, structure and, to a somewhat lesser extent, autonomy-supportive teacher behavior are scored the highest. In addition, the table reveals that there is some variation in what students perceive in their classes with regard to the behavior of their teachers. Furthermore, it is clear that, on average, the academic engagement of students decreases and their procrastination behavior increases even after about 2 months in secondary education, comparable to a small effect size (if this trend continues in a linear manner during the school year, then the decrease of academic engagement and increase of procrastination behavior would be comparable to a large effect size).

### Main Analysis

Multilevel analyses revealed that both need-supportive and need-thwarting teacher behaviors can explain the differences (and changes) in students’ academic engagement and procrastination behavior (see Tables 2, 3). Remarkable is that all dimensions of need-supportive teacher behavior better explained the differences and changes in academic engagement (which can be considered as an adaptive form of student functioning), while all need-thwarting teacher behavior dimensions were a better explanation for procrastination behavior (which can be considered as a form of maladaptive student functioning). For example, the need-supportive teaching behavior structure explained (on its own) 16% of the total variance in academic engagement, while the need-thwarting teacher behavior characterized by chaos, uncertainty, and inconsistency in the classroom explained 9% of the total variance in academic engagement. With regard to procrastination behavior, the need-thwarting behavior dimensions explained (on their own) between 6 and 13% of the variance, while the need-supportive dimensions explained between 2 and 7% of the total variance.

**TABLE 2:** Summarizing overview of a selection of the results of the multilevel analyses with effects of teacher behavior and student gender on students’ academic engagement.

Academic engagement					
**Effect of need-supporting teacher behavior^a^**			**Effect of need-thwarting teacher behavior^a^**		
	**Coefficient**	**SE**		**Coefficient**	**SE**

Autonomy support	0.372***	0.066	Teacher thwart—control	−0.291***	0.072
Student gender (0 = boy, 1 = girl)	−0.029	0.065	Student gender (0 = boy, 1 = girl)	−0.037	0.066
Interaction effect	−0.177*	0.063	Interaction effect	0.088	0.106

Structure	0.468***	0.064	Teacher thwart—chaos/inconsistency	−0.389***	0.076
Student gender (0 = boy, 1 = girl)	0.011	0.062	Student gender (0 = boy, 1 = girl)	−0.021	0.065
Interaction effect	−0.152*	0.063	Interaction effect	0.166	0.111

Teacher involvement	0.403***	0.069	Teacher thwart—neglect/rejection	−0.347***	0.068
Student gender (0 = boy, 1 = girl)	−0.017	0.064	Student gender (0 = boy, 1 = girl)	0.002	0.065
Interaction effect	−0.196**	0.101	Interaction effect	0.171*	0.098

^*a*^All effects are controlled for academic engagement at the start of the school year. *p < 0.10, **p < 0.05, ***p < 0.01 (two-sided testing).

**TABLE 3:** Summarizing overview of a selection of the results of the multilevel analyses with effects of teacher behavior and student gender on students’ procrastination behavior.

Procrastination behavior					
**Effect of need-supporting teacher behavior^a^**			**Effect of need-thwarting teacher behavior^a^**		
	**Coefficient**	**SE**		**Coefficient**	**SE**

Autonomy support	−0.228***	0.070	Teacher thwart—control	0.363***	0.074
Student gender (0 = boy, 1 = girl)	0.019	0.069	Student gender (0 = boy, 1 = girl)	0.026	0.067
Interaction effect	0.067	0.098	Interaction effect	−0.033	0.108

Structure	−0.242***	0.071	Teacher thwart—chaos/Inconsistency	0.339***	0.080
Student gender (0 = boy, 1 = girl)	−0.018	0.068	Student gender (0 = boy, 1 = girl)	0.007	0.068
Interaction effect	−0.001	0.102	Interaction effect	−0.021	0.117

Teacher involvement	−0.203**	0.073	Teacher thwart—neglect/rejection	0.277***	0.072
Student gender (0 = boy, 1 = girl)	−0.006	0.069	Student gender (0 = boy, 1 = girl)	−0.025	0.069
Interaction effect	−0.110^*b*^	0.108	Interaction effect	−0.160^*b*^	0.098

^*a*^All effects are controlled for procrastination at the start of the school year. ^*b*^Additional analyses indicated that the effect of need-supportive/need-thwarting teacher behavior (teacher involvement/teacher thwart—neglect/rejection) was not significant for girls. *p < 0.10, **p < 0.05, ***p < 0.01 (two-sided testing).

In addition, providing structure seemed to be the most important need-supportive teacher behavior dimension in relation to students’ academic engagement, followed by teacher involvement and autonomy support. In the same vein, teacher’s need-thwarting behavior characterized by chaos, uncertainty, and/or inconsistency in the classroom had the strongest negative effect of the need-thwarting teacher behavior dimensions on students’ academic engagement, followed by teacher behavior characterized by neglect and rejection and a controlling teacher behavior. Additional analyses revealed that the need-thwarting dimensions of teacher behavior had significant unique effects on top of the (unique and joint) effects of need-supporting dimensions of teacher behavior, with the exception of the effect of need-thwarting teacher behavior characterized by chaos, uncertainty, and inconsistency in the classroom in relation to the effect of delivering structure.

Furthermore, evidence was found for differential effects of (mainly) need-supportive behavior in relation to student gender indicating that boys are, compared to girls, more sensitive to the positive effects of need-supportive teacher behavior with regard to their academic engagement. The differences in sensitiveness between boys and girls were most pronounced for teacher involvement (and need-thwarting teacher behavior related to neglect and rejection).

A somewhat different picture arose with regard to procrastination behavior. A controlling teacher behavior as well as teacher behavior characterized by chaos, uncertainty, and inconsistency in the classroom were the most strongly positively related to the differences (and changes) in procrastination behavior of students, indicating that students procrastinated more during the school year when their teachers exhibited a controlling behavior and created learning environments with a lot of chaos and uncertainty and behaved inconsistently toward their students. Teacher’s neglect or rejection of students mattered as well, although to a somewhat lesser extent. The need-supportive teacher behavior dimensions operated more or less in the same vein, albeit that their effect was in the opposite direction. Additional analyses revealed that the need-supportive dimensions of teacher behavior had no significant unique effects on top of the (unique and joint) effects of the need-thwarting dimensions of teacher behavior, with the exception of the effect of delivering structure. This indicates that delivering structure by the teacher might operate as a protective factor with regard to (the development of) students’ procrastination behavior.

Differential effects of the need-thwarting teacher behaviors on students’ procrastination in relation to student gender did almost not exist. However, an exception was found with regard to teacher neglect/rejection: additional in-depth analysis revealed that the positive effect of teacher neglect/rejection and the negative effect of teacher involvement on students’ procrastination behavior for girls were not significant, indicating (again) that boys were, compared to girls, more sensitive to teachers’ involvement and teachers’ neglect/rejection.

## Conclusion and Discussion

The aim of this study was to investigate whether need-supportive and need-thwarting teacher behaviors (as perceived by students) were related to students’ academic engagement and procrastination behavior, which can be respectively conceived as a form of adaptive and maladaptive student behavior. In addition, the possibility of differential effects of need-supportive and need-thwarting teacher behaviors in relation to student gender was explored. The study was quite innovative since attention was paid to the need-supportive as well as need-thwarting behaviors within the same study, forms of adaptive as well as maladaptive student behaviors were the subject of the research, attention was paid to differential effects of the mentioned teacher behaviors, and a longitudinal approach was adopted.

Based on the SDT, and in particular the basic psychological needs theory (and in agreement with the self-system model of motivational development), it was expected that the different dimensions of need-supportive and need-thwarting teacher behavior were associated with students’ academic engagement and procrastination behavior. In line with the (small amount of) literature on differential effects of learning environment characteristics in relation to student gender, it was expected that, if differential effects would be found, they would indicate a higher sensitivity of boys toward the behavior of their teachers.

### Main Conclusions

In line with the expectations and the literature on need-supportive and need-thwarting conditions (Vansteenkiste et al., 2020), evidence was found for the significant effects of all considered need-supportive as well as need-thwarting teacher behavior dimensions on students’ academic engagement and procrastination behavior. Furthermore, the findings indicated that need-supportive teacher behavior was most important for academic engagement (which was conceived as a form of adaptive behavior), while need-thwarting teacher behavior was most important for procrastination behavior (which was conceived as a form of maladaptive behavior). This finding is in agreement with the study of Skinner et al. (2008), in which the need-supportive dimensions had stronger effects on the engagement dimensions compared to the disaffection dimensions (maladaptive functioning), and with the dual-process model and findings of Jang et al. (2016). In addition, in three cases out of six, need-supportive as well as need-thwarting teacher behaviors had, together, unique effects on the student outcome studied, which delivers evidence that it is worthwhile distinguishing between and taking into account need-supportive as well as need-thwarting teacher behaviors when studying adaptive and maladaptive student behavior (which is in agreement with recent views on the BPNT; see, e.g., Bartholomew et al., 2011; Vansteenkiste et al., 2020;, and the dual-process model as described by Jang et al., 2016). Furthermore, the fact that some dimensions of need-thwarting teacher behavior had an effect on students’ behavior above and beyond the related need-supportive teacher behavior dimensions suggests that need frustration does yield, in some cases, additional functional costs, as Vansteenkiste et al. (2020) recently mentioned.

Furthermore, in line with the literature on differential effects related to student gender, evidence was found for some differential effects of teacher behavior, all pointing to a higher sensitivity of boys. While for all dimensions of need-supportive teacher behavior differential effects were found in relation to students’ academic engagement, it was also found for teacher involvement related to students’ procrastination behavior and teacher neglect/rejection in relation to students’ academic engagement and procrastination behavior. However, while boys seemed to be more sensitive, compared to girls, with respect to teacher involvement and teacher neglect/rejection in relation to their academic engagement, only boys seemed to be sensitive to teacher involvement and teacher neglect/rejection in relation to procrastination behavior. Combined, these findings suggest that teacher involvement and teacher neglect/rejection are relevant factors for students’ (evolution in) academic engagement (even more so for boys) and seem to be a protective, respectively, thwarting factor in the development of procrastination behavior of boys. A possible explanation for the greater (or solely) effect of teacher involvement and teacher neglect/rejection on boys could be that boys are more triggered by a smile and encouragement of the teacher because they are often more externally motivated than girls (while girls are often more internally/intrinsically/autonomous motivated than boys; see, e.g., Opdenakker et al., 2012). The SDT posits that supporting the need for relatedness (or thwarting this need) is relevant for the so-called internalization process, by which values and regulations are “taken in” and non-intrinsically motivated behaviors can become self-determined; it concerns, for example, the internalization of the regulation of positive school-related behaviors (Ryan and Deci, 2000). There is some evidence and explanation in the research of Fan (2011). Fan’s (2011) study revealed that the quality of teacher–student relationship (warmth and encouragement) was only for boys related to their utility value (which captures more extrinsic reasons for doing a task in contrast to intrinsic value), and utility value was somewhat more for boys than for girls related to academic engagement. Another explanation might be that teacher involvement, neglect, and rejection are more important for boys because they have a higher exposure to this kind of teacher behavior than girls. There is some evidence that teachers are, on average, more involved with their male students, offering them more acknowledgment, encouragement, approval, criticism, and corrective feedback than their female students (Brophy and Good, 1974; Meece et al., 2006). Furthermore, according to Brophy and Good (1974), teachers communicate in this way different learning expectations for their male and female students.

For students’ academic engagement, the findings show that, for all students (but even more for boys), in particular giving structure and also being involved with students and being autonomy-supportive, thus being supportive of the needs of competence, relatedness, and autonomy, have stimulating potential, while need-thwarting teacher behaviors such as neglecting or rejecting students and exhibiting a controlling behavior have harmful potential.

For students’ procrastination behavior, the results indicate that, for all students, a controlling teacher behavior and being in learning environments in which there is much chaos, uncertainty, and inconsistent teacher behavior—thus being with teachers who exhibit need-thwarting behaviors—have harmful potential, meaning evoking procrastination. In contrast, being with teachers who deliver structure has potential to diminish the procrastination behavior. These findings are in line with the (theoretical) work of Vermunt and Verloop (1999) on the congruence and friction between learning and teaching. Vermunt and Verloop (1999), who make a plea for process-oriented teaching, indicate that, in case student self-regulation is low (which is also the case when students procrastinate since procrastination is nowadays often seen as a self-regulation failure; Pychyl and Flett, 2012), it is important that teachers help students with the regulation (of learning processes) by teacher-initiated or shared-controlled teaching strategies in order to create congruence or constructive frictions. This shows similarity with teachers scoring high on structure while in the mean time being autonomy-supportive. According to Vermunt and Verloop (1999), constructive frictions may be necessary “to make students willing to change and to stimulate them to develop skill in the use of learning and thinking activities they are not inclined to use on their own” (p. 270). However, destructive frictions, which occur when the degree of students’ self-regulation is inadequate and the teacher regulation is deficient too (which resembles teachers scoring high on the creation of an environment in which chaos, uncertainty, and inconsistent teacher behavior are present) may cause a decrease in the learning or thinking skills of students. Lastly, for boys, also teacher involvement seems to have unique potential to diminish the procrastination behavior, while teachers’ neglect or even rejection of boys has the potential to evoke or increase the procrastination behavior. The results with regard to the importance of structure and the need-thwarting behaviors of chaos, uncertainty, and inconsistency are in line with the scarce literature on contextual and social environment factors (including teacher/instructor factors) pointing to the positive effects of instructor support and organization (e.g., works of Grunschel et al., 2013 and Corkin et al., 2014). However, the findings of the negative effect of a controlling teacher behavior on evoking/increasing procrastination behavior are rather new to the knowledge base on procrastination behavior related to school.

In summary, our findings are in line with the SDT (in particular the sub-theory BPNT) and the related self-system model of motivational development, in which the importance of the basic psychological needs of competence, autonomy, and relatedness is stressed in relation to students’ adaptive and maladaptive functioning. In addition, our findings deliver evidence for the dual-process model, and our findings of the importance of structure (for students’ academic engagement and procrastination) and the harmful effects of chaos, inconsistency, and uncertainty (evoking procrastination), which are supposed to be of relevance for students’ need to feel competent (otherwise stated to feel self-efficacious), are in agreement with Steel’s temporal motivation theory (Steel, 2007). Our findings regarding the higher sensitivity of boys in relation to the effects of teacher behavior are in line with the scarce literature on this topic.

The present study was among the first to investigate, within the same study, the three dimensions of need-supportive as well as the three dimensions of need-thwarting teacher behavior in line with SDT-BPNT, to study these teacher behaviors while adopting a longitudinal design, to link the mentioned teacher behaviors with an adaptive form of student behavior (academic engagement) as well as a maladaptive form of student behavior (procrastination behavior), and to pay attention to possible differential effects of teacher behavior in relation to student gender. The results revealed that looking at teacher behaviors from a motivational perspective, and in particular from the viewpoint of supporting and thwarting basic psychological needs, is a fruitful way to gain insights into the emergence and development of students’ academic engagement and procrastination behavior. In addition, the findings indicated that it is important not only to look at need-supportive teacher behavior but also to investigate need-thwarting teacher behavior when students’ behavior is the focus of the research and that it is worthwhile to look, in particular, at need-thwarting teacher behavior when the emergence of maladaptive student behavior is focused on. This is in line with the ideas of the dual-process model as described by Jang et al. (2016). Furthermore, the study delivered evidence that student gender sometimes operates as a moderator in teacher behavior–student behavior associations, which has not only scientific implications but practical implications as well.

### Limitations and Suggestions for Further Directions

Although the study revealed important results and expands the current knowledge and evidence on the effects of teacher behavior on boys’ and girls’ academic engagements and procrastination behaviors related to school, the study also has some limitations.

One of the limitations of this study is linked to the reliance on student perceptions of teacher behavior and self-reports of academic engagement and procrastination behavior. Student perceptions of their teachers’ behavior, and of their learning environment in general, are seen as very valuable and convenient in motivational and learning environment research, and Kulik (2001) concludes in his review on the validity of student ratings that they have high validity (strong correlation with classroom observations and expert observations). Also, from a SDT perspective, student perceptions and students’ self-reports are seen as important: “SDT sees them as important tools for assessing the functional significance and meaning of events, and as having a critical role within motivation sciences alongside other methods” (Ryan and Deci, 2020, p. 8). However, using student ratings might have inflated the obtained relationships with students’ academic engagement and procrastination behavior. The use of observational data would overcome this particular problem; however, these kinds of data have their own shortcomings as well.

Furthermore, the study was based on the measurement of student outcomes at two time points. Although this is already an improvement to much research in which all variables are measured at the same time, it would be interesting to study the effects of teachers’ need-supportive and need-thwarting behaviors within a longitudinal design with more than two time points (and covering a longer time span, for example a whole school year), paying attention to changes in teacher behavior and student outcomes and to reciprocal effects between them.

All in all, the findings of the present study contribute to our growing understanding of the facilitating and undermining factors of students’ adaptive and maladaptive functioning in relation to learning tasks at school and highlight the importance of both teachers’ need-supportive and need-thwarting behaviors in daily classrooms. In addition, the study contributes to deepen our insight into and understanding of what kind of teacher behavior affects boys’ and girls’ behaviors equally and differently and underscores the evidence that a “one-size-fits-all” approach is too simplistic (see, e.g., Blumenfeld et al., 2005; Rosenzweig and Wigfield, 2016) to stimulate the academic engagement and diminish the procrastination behavior of boys and girls equally. As such, the findings pose a challenge to various educational effectiveness models and motivation theories. In a recent article of Vansteenkiste et al. (2020), the importance of moderators such as socio-demographics in relation to SDT is recognized.

In line with this, further research should also explore the effects of teacher behavior, and in particular the effects of need-supportive and need-thwarting teacher behaviors, on the development of the capacities and motivation for self-regulated learning and prosocial behavior toward peers (while taking into account the possibility of differential effects with regard to boys and girls and other relevant student characteristics) since these are enduring motivational resources to which students’ academic engagement and active participation in school likely positively contribute and that may act as protective buffers as students go through challenging transitions in school and life.

Finally, additional directions for future research could also be the inclusion of perspectives of multiple informants concerning teachers’ behavior, for example, the perspectives of teachers themselves and independent observers, as well as the inclusion of contextual factors such as type of school.

## Data Availability Statement

The raw data supporting the conclusions of this article will be made available by the authors, without undue reservation.

## Ethics Statement

Ethical review and approval was not required for the study on human participants in accordance with the local legislation and institutional requirements. Written informed consent to participate in this study was provided by the participants’ legal guardian/next of kin.

## Author Contributions

The author designed the study, was in charge of the data collection procedure, analyzed the data, and wrote the manuscript.

## Conflict of Interest

The author declares that the research was conducted in the absence of any commercial or financial relationships that could be construed as a potential conflict of interest.
